# A STOP‐Gain RNF213 Variant Causes Chorea, Stroke‐Like Episodes, and Leigh Syndrome‐Like Encephalopathy

**DOI:** 10.1002/mds.70077

**Published:** 2025-09-26

**Authors:** Roberta Bovenzi, Mariasavina Severino, Jennifer Nichols, Fred Shen, Ignacio J. Keller Sarmiento, Bernabe I. Bustos, Lisa Kinsley, Dimitri Krainc, Niccolò E. Mencacci

**Affiliations:** ^1^ Ken and Ruth Davee Department of Neurology and Simpson Querrey Center for Neurogenetics Northwestern University, Feinberg School of Medicine Chicago Illinois USA; ^2^ Department of Systems Medicine University of Tor Vergata Rome Italy; ^3^ Neuroradiology Unit IRCCS Institute Giannina Gaslini Genoa Italy

*RNF213* (OMIM *613768), the primary susceptibility gene for moyamoya disease (MMD), encodes an E3 ubiquitin ligase involved in angiogenesis, lipid metabolism, and blood flow regulation.^1^ Recently, it has been recognized as a Mendelian disease gene, with a spectrum including Leigh syndrome, a mitochondrial encephalopathy with characteristic magnetic resonance imaging (MRI) abnormalities.^2^

Here, we report two related individuals harboring a novel *RNF213* variant with distinctive clinical features, including stroke‐like episodes, Leigh syndrome‐like neuroradiological findings, and late‐onset progressive generalized chorea in one subject.

Case 1 is a 68‐year‐old man with history of autism spectrum disorder and stroke‐like episodes featuring headache, confusion, and speech difficulties, initially diagnosed as complex migraine. In his mid‐50s, he developed slowly progressive gait imbalance and involuntary choreic movements, memory/behavioral changes, and slurred speech. No episodes of acute worsening of hyperkinetic disorders were reported. An extensive workup for acquired and genetic causes of chorea and ataxia was unremarkable. A brain MRI performed at age 59 years showed T2/FLAIR hyperintensities within the central pons and midbrain, patchy putaminal/caudate changes with atrophy, periventricular white matter loss, and global atrophy (Fig. 1B). Brain and neck magnetic resonance angiography (MRA) were unremarkable.

**FIG. 1:**
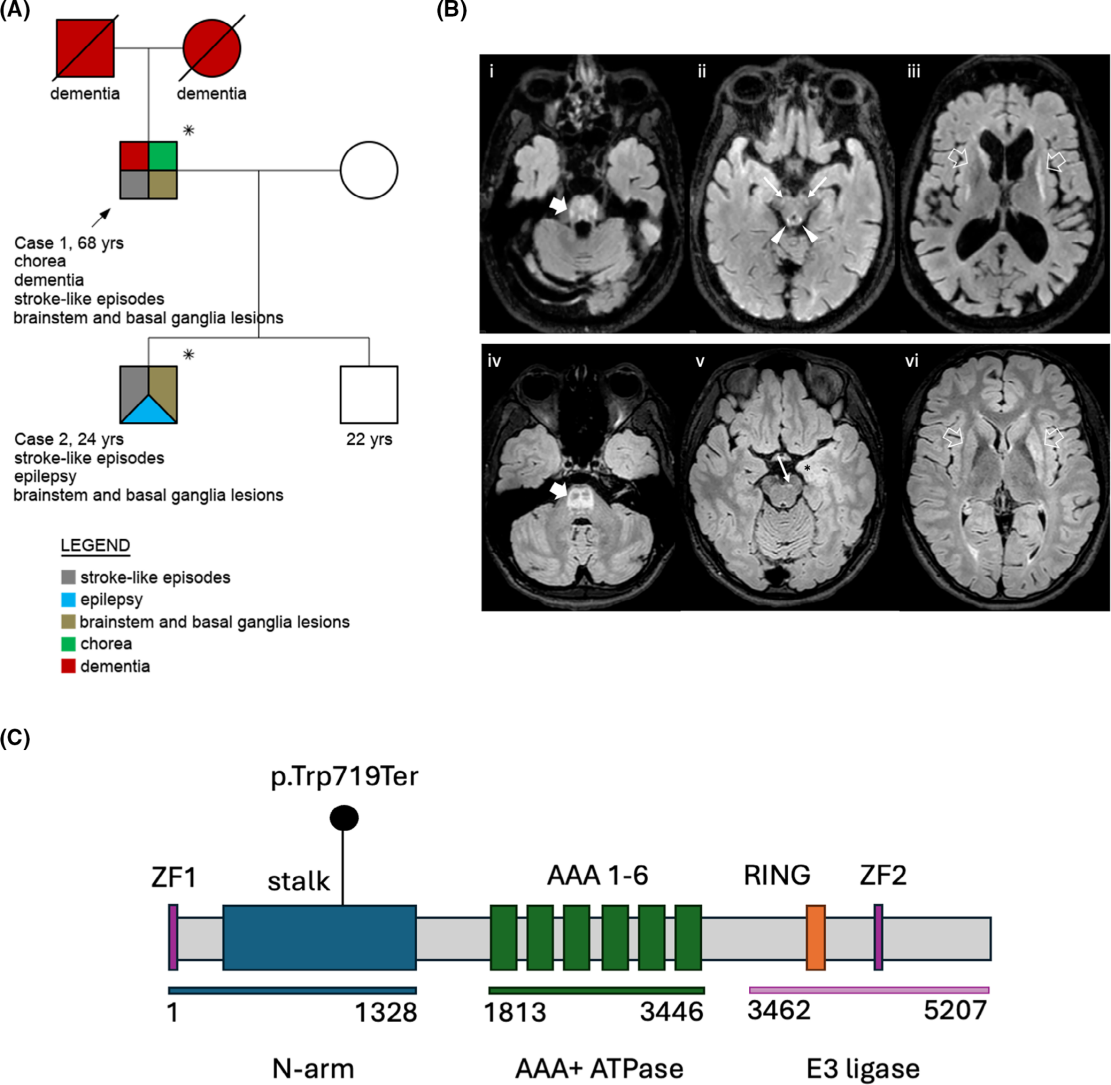
(A) Family tree; *subjects who underwent genetic testing. (B) Brain magnetic resonance imaging (MRI) of Case 1 at age 63 years (Bi‐iii) and Case 2 at age 16 years (Biv‐vi). Bi, Biv: pontine T2/FLAIR hyperintensities (thick arrows). Bii, Bv: involvement of cerebral peduncles (thin arrows) and midbrain tectum (arrowheads). Biii, Bvi: putamen and caudate nuclei atrophy with asymmetric/patchy signal alterations (empty arrows). (C) Diagram of *RNF213* gene illustrating the STOP‐gain variant (adapted from Mineharu et al.^5^). [Color figure can be viewed at wileyonlinelibrary.com]

Neurological examination at age 64 years showed dysarthria, continuous generalized chorea (right predominant), and mild bilateral dysmetria (see Video S1).

Case 2, his 24‐year‐old son, experienced starting at age 12 years episodes of headache, aphasia, and right limb numbness, diagnosed as complex migraine. An initial brain MRI showed strikingly similar abnormalities to his father's MRI, including small T2/FLAIR hyperintensitities and T1 hypointensities in the central pons, faint symmetrical T2/FLAIR hyperintensities in the putamen and caudate, and slight bilateral putaminal size reduction. Months later, he developed acute encephalopathy, and a repeated brain MRI revealed worsening basal ganglia lesions without contrast enhancement or restricted diffusion. Brain MRA was normal. Cerebrospinal fluid and serum inflammatory, infectious, and metabolic workup were unremarkable. Electroencephalogram showed bilateral frontotemporal slowing and left frontotemporal epileptiform activity.

Following discharge, he developed focal epilepsy with secondary generalization, partially controlled with lamotrigine. At age 17 years, MRI revealed progression of pontine alterations, new midbrain lesions, and slight accentuation of left putaminal changes (Fig. 1B). Magnetic resonance spectroscopy with lactate peak was normal. At age 21 years, only subtle dysarthria and tandem gait difficulties persisted.

Whole exome sequencing in both individuals revealed a novel heterozygous *RNF213* variant (ENST00000319921.4:c.2157G>A; p.Trp719*; Fig. 1C), as the most likely genetic cause for the family phenotype. Full genetic data are presented in File S1.

The two cases showed different neurological presentations but a strikingly similar neuroimaging phenotype, with selective central pontine and dorsal tegmental involvement sparing corticospinal tracts, focal/confluent signal putaminal‐caudate lesions with volume loss, and cerebral peduncles involvement.

These neuroradiological features resemble Leigh syndrome, which has been recently described in three children with heterozygous *RNF213* variants.^2^ Previously reported *RNF213* variants were de novo in two cases and inherited from a mother with cerebral hemorrhage in one,^2^ indicating variable expressivity and inheritance patterns.^1^

The present variant is predicted to cause truncation upstream of the RING finger and ATPase domains. Although *RNF213* is loss‐of‐function (LOF)‐tolerant (pLI = 0.00), LOF variants have been associated with intracranial aneurysms and MMD,^1^ and may increase vascular permeability and proliferation.^3^, ^4^ This report expands the *RNF213* phenotypic spectrum, linking a STOP‐gain variant to a novel phenotype with variable presentation and characteristic MRI findings. *RNF213* variants should be considered in the differential of cases with adult‐onset progressive generalized chorea, especially if brain MRI shows features reminiscent of Leigh syndrome.

## Author Roles

(1) Research Project: A. Conception and Design, B. Organization, C. Data Acquisition; (2) Statistical Analysis: A. Design, B. Execution, C. Review and Critique; (3) Manuscript Preparation: A. Writing of the First Draft, B. Revision for Intellectual Content, C. Approval of the Final Version.

R.B.: 1A, 1C, 3A, 3B, 3C.

M.S.: 1A, 1C, 3A, 3B, 3C.

J.N.: 1C, 3B, 3C.

F.S.: 1C, 3A, 3B, 3C.

I.J.K.S.: 1C, 3B, 3C.

B.I.B.: 1C, 3B, 3C.

L.K.: 1C, 3B, 3C.

D.K.: 1C, 3B, 3C.

N.E.M.: 1A, 1C, 3A, 3B, 3C.

## Financial Disclosures of All Authors

I.J.K.S. is supported by the Align Science Across Parkinson's (ASAP) Global Parkinson's Genetics Program (GP2). D.K. is the Founder and Scientific Advisory Board Chair of Lysosomal Therapeutics Inc. and Vanqua Bio. D.K. serves on the scientific advisory boards of The Silverstein Foundation, Intellia Therapeutics, AcureX, and Prevail Therapeutics and is a Venture Partner at OrbiMed. N.E.M. receives National Institutes of Health (NIH) funding (1K08NS131581) and is supported by the Align Science Across Parkinson's (ASAP) Global Parkinson's Genetics Program (GP2). N.E.M. is a member of the steering committee of the PD GENEration study for which he receives an honorarium from the Parkinson's Foundation.

## Supporting information


**Data S1.** Supporting Information.

**Video S1.** Case 1. Choreic movements in the neck, trunk, and upper limbs, more prominent on the right side. Impaired rapid alternating movements in the four extremities with mild dysmetria on finger‐to‐nose bilaterally. Gait shows hyperkinetic features, reduced left arm swing, slow turns, and a mildly widened base.

## Data Availability

The data that support the findings of this study are available from the corresponding author upon reasonable request.
