# Supplementary material for: A STOP‐Gain RNF213 Variant Causes Chorea, Stroke‐Like Episodes, and Leigh Syndrome‐Like Encephalopathy
Source: Mov Disord. 2025 Sep 26;41(1):262–4. doi: 10.1002/mds.70077 (PMC12882026; doi:10.1002/mds.70077)
Supplement: Supplementary file 1 — Data S1. Supporting Information. [file MDS-41-262-s001.docx]

**Supplementary File S1**

This study involved two individuals from an American family of European ancestry evaluated at the Movement Disorders Genetics Clinic of Northwestern Medicine, Chicago. **Figure 1A,** shows the family pedigree. Repeat expansion analyses for HD, SCA1,2,3,6,7,8,17, DRPLA and *c9orf72* were performed with Expansion Hunter^1^ in Case 1. Research-based short-read whole-exome sequencing (WES) was performed in the two individuals as previously described^2,3^. Mitochondrial DNA was not analyzed due to the suspected paternal-son inheritance pattern. Variants were filtered and prioritized based on minor allele frequency (<0.0001) in the Genome Aggregation Database (gnomAD) v4.1.0(https://gnomad.broadinstitute.org/), and *in silico* prediction scores^2,3^.

Repeat expansion analyses were negative in Case 1. WES revealed 42 shared rare high and moderate impact heterozygous variants, none of which in genes associated with chorea, epilepsy, mitochondrial, peroxisomal, or metabolic disorders. Six variants were loss-of-function (LOF) and 36 missense.

Using criteria of frequency and protein impact, a novel heterozygous variant in *RNF213* ENST00000319921.4:c.2157G>A; (p.Trp719*) (NM_001256071.1) was prioritized as the best candidate.

The variant was predicted to generate a truncated protein product upstream to the AAA+ adenosine triphosphatase (ATPase) and the Really Interesting New Gene (RING) domains (**Figure 1C**). The variant is unreported in gnomAD v4.1.0, the Human Gene Mutation Database (http://www.hgmd.cf.ac.uk), and ClinVar (<https://www.ncbi.nlm.nih.gov/clinvar/>).

According to the American College of Medical Genetics (ACMG) criteria, it was classified as Variant of Uncertain Significance (PM2, PVS1, PP3).

**References**

1. Dolzhenko E, Deshpande V, Schlesinger F, et al. ExpansionHunter: A sequence-graph-based tool to analyze variation in short tandem repeat regions. *Bioinformatics*. 2019;35(22). doi:10.1093/bioinformatics/btz431

2. Carecchio M, Invernizzi F, Gonzàlez-Latapi P, et al. Frequency and phenotypic spectrum of KMT2B dystonia in childhood: A single-center cohort study. *Movement Disorders*. 2019;34(10). doi:10.1002/mds.27771

3. Keller Sarmiento IJ, Bustos BI, Blackburn J, et al. <scp> De novo *FRMD5* </scp> Missense Variants in Patients with Childhood‐Onset Ataxia, Prominent Nystagmus, and Seizures. *Movement Disorders*. 2024;39(7):1231-1236. doi:10.1002/mds.29791
